# First genomic evidence and molecular epidemiology of porcine bufavirus in Myanmar: Whole-genome characterization, phylogenetic insights, and potential zoonotic implications

**DOI:** 10.14202/vetworld.2025.4157-4171

**Published:** 2025-12-31

**Authors:** Hnin Wai Phyu, Kamonpan Charoenkul, Chanakarn Nasamran, Kitikhun Udom, Eaint Min Phyu, Yu Nandi Thaw, Supassama Chaiyawong, Thant Nyi Lin, Min Thein Maw, Alongkorn Amonsin

**Affiliations:** 1 Center of Excellence Emerging and Re-emerging Infectious Diseases in Animals, Chulalongkorn University, Bangkok, 10330, Thailand; 2 Department of Veterinary Public Health, Faculty of Veterinary Science, Chulalongkorn University, Bangkok, 10330, Thailand; 3 Department of Veterinary Public Health, University of Veterinary Science, Yezin, Nay Pyi Taw, Myanmar; 4 Livestock Breeding and Veterinary Department, Ministry of Agriculture, Livestock and Irrigation, Nay Pyi Taw, Myanmar

**Keywords:** emerging swine viruses, molecular detection, Myanmar pig farms, One Health surveillance, phylogenetic analysis, porcine bufavirus, whole-genome characterization, zoonotic potential

## Abstract

**Background and Aim:**

Porcine bufavirus (PBuV) is an emerging enteric parvovirus increasingly reported in swine populations worldwide, but its epidemiological and genomic characteristics remain poorly understood in Southeast Asia. This study aimed to conduct a cross-sectional survey to determine the occurrence of PBuV in pig farms in Myanmar and to genetically characterize circulating Myanmar-PBuVs using whole-genome sequencing (WGS).

**Materials and Methods:**

Between January and September 2023, 445 rectal swab samples were collected from pigs of various age groups and clinical statuses across 19 pig farms in the Yangon and Nay Pyi Taw Regions. Samples were screened using nested polymerase chain reaction (PCR) targeting the nonstructural protein 1 (*NS1*) gene. Seven PCR-positive samples were selected for WGS based on farm location, animal age, collection time, and amplicon quality. Phylogenetic analyses of whole genomes and *NS1*, viral protein 1 (*VP1*), and viral protein 2 (*VP2*) genes were performed using maximum–likelihood methods. Nucleotide and amino acid identities, conserved motifs, and unique mutations were assessed to determine genetic relationships with global PBuV and bufavirus (BuV) lineages.

**Results:**

PBuV positivity was 15.06% (67/445; 95% confidence interval: 11.9–18.7), with detection in both diarrheic and healthy pigs. Fattening pigs exhibited the highest positivity (36.55%), and PBuV occurrence was significantly associated with winter months (p < 0.05). Seven Myanmar-PBuVs were successfully sequenced and clustered within the PBuV clade, showing close genetic relatedness to Austrian and Chinese PBuVs. Myanmar-PBuVs shared 91.81%–100% whole-genome nucleotide identity, with substantially lower identity (48%–63%) to BuVs from humans, dogs (*Canis lupus familiaris*), bats (various species), and rats (*Rattus* spp.). Conserved *NS1*, *VP1*, and *VP2* motifs were preserved; however, unique amino acid insertions in *NS1* (notably in CU34347) and several *VP2* substitutions suggested potential region-specific evolution.

**Conclusion:**

This study provides the first genomic evidence of PBuV circulation in Myanmar and expands the global PBuV sequence database. The high detection in fattening pigs, seasonal trends, and phylogenetic proximity to European and Chinese strains highlight possible transboundary introduction pathways. Genetic similarities between Myanmar-PBuVs and human BuV in *VP1*/*VP2* underscore the importance of One Health surveillance. Broader-scale longitudinal studies are needed to clarify PBuV evolution, disease association, and zoonotic potential.

## INTRODUCTION

Bufavirus (BuV) is an emerging enteric pathogen classified under the family *Parvoviridae* and the genus *Protoparvovirus*. It is a non-enveloped, non-segmented, linear, single-stranded DNA virus that infects a wide range of avian and mammalian species [1]. Its genome is relatively small (4–6 kb) and encodes nonstructural protein 1 (NS1), structural proteins viral protein 1 (VP1) and viral protein 2 (VP2), and a small hypothetical protein [2, 3]. The corresponding genes, *NS1*, *VP1*, and *VP2*, are key targets for molecular detection and phylogenetic analysis. BuV was first identified in 2012 in children with acute diarrhea in Burkina Faso [4]. Since then, human BuV infections have been documented in both children and adults in several countries, including Bhutan, China, France, Finland, the Netherlands, South Africa, Thailand, and Turkey [3–9]. Beyond human cases, BuV has been detected in multiple animal hosts, such as dogs (*Canis lupus familiaris*), pigs (*Sus scrofa domesticus*), bats, cats (*Felis catus*), rats (*Rattus* spp.), shrews, and non-human primates, highlighting its broad host range [2, 10–16]. Notably, BuV has been reported in dogs from China, Thailand, and Turkey [10, 11, 17], as well as in cats in China and hedgehogs in Italy [18, 19].

Porcine bufavirus (PBuV) was first identified in pigs in Hungary in 2016. Subsequent studies have reported PBuV in pigs presenting with diarrhea, posterior paraplegia, and in clinically normal animals [20]. Genetic analyses from China revealed that the VP1 and VP2 proteins of PBuVs share close similarity with those of human BuVs, suggesting a potential zoonotic risk [21]. Correspondingly, the *VP1* and *VP2* genes exhibit high sequence similarity between PBuVs and human BuVs. Although PBuV has been detected in both diarrheic and non-diarrheic pigs, its exact pathogenic role and association with gastrointestinal disease remain unresolved. Furthermore, PBuV frequently co-occurs with other swine enteric viruses, including porcine epidemic diarrhea virus, porcine deltacoronavirus, porcine rotavirus, and porcine circovirus 2, which may contribute to mixed infections and more severe clinical outcomes [22, 23].

Despite the growing recognition of BuV as an emerging enteric pathogen in both humans and animals, information on PBuV remains limited, particularly in Southeast Asia. Most available studies originate from Europe and China, where PBuV has been detected in pigs with varying clinical presentations and shown to possess genetic features closely related to human BuVs [20–23]. However, substantial knowledge gaps persist regarding the epidemiology, genetic diversity, host range, and potential zoonotic significance of PBuV in regions with high pig population density and active cross-border livestock movement. In Myanmar, where pig-farming systems vary widely in scale and biosecurity practices, no prior studies have characterized the presence, distribution, or genomic profiles of PBuV. Moreover, the scarcity of complete PBuV genome sequences in global databases hinders comprehensive phylogenetic comparisons and limits understanding of viral evolution. The contribution of PBuV to gastrointestinal disease in pigs, its co-circulation with other enteric pathogens, and its possible spillover risk remain poorly understood. These gaps underscore the need for systematic molecular surveillance and whole-genome-based characterization to provide foundational epidemiological and genomic insights.

This study aimed to investigate the presence and distribution of PBuV in pig farms in Myanmar and to provide the first genomic characterization of Myanmar-PBuV strains. Specifically, the study sought to (i) detect PBuV in pigs across different age groups, clinical conditions, and farm types using nested polymerase chain reaction (PCR) targeting the *NS1* gene; (ii) generate and analyze whole-genome sequences of Myanmar-PBuVs to assess their genetic diversity; (iii) determine phylogenetic relationships between Myanmar-PBuVs and global BuV strains from pigs, humans, and other animal hosts; and (iv) evaluate conserved and unique amino acid motifs associated with viral replication, structure, and potential host adaptation. By addressing these objectives, the study aims to enhance understanding of PBuV epidemiology in Myanmar, contribute novel genomic data to the global repository, and support One Health–oriented risk assessment for future surveillance and control programs.

## MATERIALS AND METHODS

### Ethical approval

This study was conducted in strict accordance with the ethical guidelines for the care and use of animals in research established by Chulalongkorn University and the Livestock Breeding and Veterinary Department (LBVD) of Myanmar. All animal procedures, sampling methods, and biosafety practices were reviewed and approved by the Institutional Animal Care and Use Committee of the Faculty of Veterinary Science, Chulalongkorn University, under protocol number CU-VET IACUC #2331099. The study design complied with the Animal Research: Reporting of *In Vivo* Experiments (ARRIVE) 2.0 guidelines, the OIE (World Organization for Animal Health) standards for animal welfare, and national regulations governing research involving livestock.

Prior to sample collection, permission was obtained from the LBVD authorities in both Yangon and Nay Pyi Taw Regions. Farm owners were informed of the study objectives, sampling procedures, and biosafety measures, and verbal consent was obtained from all participating pig farms. No invasive procedures beyond routine rectal swabbing were performed, and all efforts were made to minimize stress and handling time for the animals. Sample collection was carried out by trained veterinarians using sterile, single-use materials to ensure animal welfare and prevent cross-contamination.

All samples were transported and processed following institutional biosafety protocols for handling potentially infectious materials. No animals were euthanized or subjected to experimental infection as part of this study. The study adhered to the principles of Replacement, Reduction, and Refinement (3Rs) to ensure the responsible use of animals in scientific research.

### Study period and location

**Figure 1:**
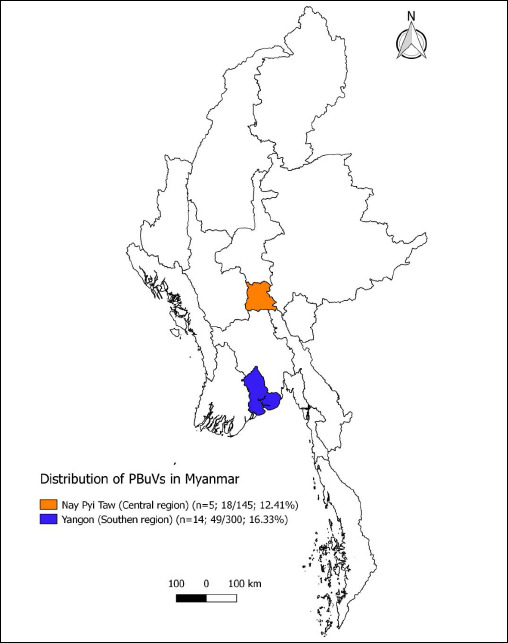
Distribution of PBuVs in Myanmar. Two sampling regions are marked, including the number of sampled pig farms, the number of PBuVs positive samples/the total number of samples, and the positive rate (Source: QGIS v3.8 Myanmar).

This study was conducted between January and September 2023 across two major pig-producing regions of Myanmar: Yangon and Nay Pyi Taw (Figure 1). Yangon, located in the southern part of the country, has a tropical monsoon climate, while Nay Pyi Taw in the central region experiences a tropical savanna climate. According to the 2018 animal census by the LBVD, pig population densities were 291,672 (28 animals/km²) in Yangon and 128,159 (18 animals/km²) in Nay Pyi Taw.

Myanmar utilizes three primary pig-farming systems, categorized by herd size: large-scale farms (100–1000 pigs, high biosecurity), medium-scale farms (10–100 pigs, moderate biosecurity and regular vaccination), and small-scale farms (1–10 pigs, limited biosecurity) [24]. Large-scale farms are mainly concentrated in Yangon, whereas medium- and small-scale farms are distributed throughout the country.

### Sample collection

A cross-sectional sampling design was employed to select 19 pig farms based on their location within high-density pig-production zones and willingness of owners to participate. A total of 445 rectal swab samples were collected from pigs of different age groups and clinical statuses, including diarrheic pigs (n = 120) and clinically healthy pigs (n = 325). Per farm visit, approximately 10–30 samples were obtained from suckling pigs (n = 65), nursery pigs (n = 215), fattening pigs (n = 145), and breeder pigs (n = 20).

Rectal swabs were collected using sterile polyester-tipped swabs inserted into the rectum, gently rotated, and placed into 2 mL of viral transport medium (Eagle Minimum Essential Medium). Samples were transported at 4°C and processed within 24 h. Upon arrival at the laboratory, 200 μL was aliquoted for DNA extraction, and remaining material was stored at –80°C. Sample information, including date of collection, age, and clinical status, was recorded.

### Detection of PBuV

For nucleic acid extraction, 200 μL of each rectal swab sample was vortexed for 15 s, followed by swab removal. The supernatant was mixed with lysis buffer and processed using the GeneAll® GENTi™ Viral DNA/RNA Extraction Kit on the GENTi™ 32 automated system (GeneAll®, Lisbon, Portugal). DNA purity (260/280 ratio of 1.8–2.0) was assessed using a Nanodrop spectrophotometer (Thermo Fisher Scientific, USA).

PBuV detection was performed using a nested PCR assay targeting the NS1 gene, employing primers modified from a previous study [22] (Supplementary Table 1). Each 25 μL PCR reaction included 3 μL DNA, HotStarTaq Master Mix (Qiagen, Germany), 0.1 μM of each primer, and distilled water. Cycling conditions for the first PCR included 95°C for 15 min, followed by 40 cycles (94°C for 30 s, 56°C for 30 s, 72°C for 1 min), and a final extension at 72°C for 10 min. The second-round PCR used identical conditions. Amplicons were visualized on 1.5% agarose gel with RedSafe staining. Previously confirmed PBuV DNA served as a positive control; nuclease-free water served as the negative control. Expected product size: 233 bp.

### Whole-genome sequencing (WGS) and genetic characterization

Seven PBuV-positive samples were selected for WGS based on farm location, sampling date, pig age, and DNA amplicon quality. Genome amplification was performed using newly designed primers (Primer3Plus) and previously published primers (Table S1). Each 50 μL PCR reaction contained 4 μL DNA, HotStartTaq Master Mix (Qiagen), 0.2 μM primers, and distilled water. PCR cycling involved an initial denaturation at 95°C for 15 min, followed by 40 cycles (94°C for 30 s, 45–50°C for 30 s, 72°C for 90 s) and a final extension at 72°C for 10 min. Amplicons were gel-purified and Sanger-sequenced (Bionic Co., Korea) using an ABI 3730xl DNA Analyzer (Applied Biosystems, USA). Sequences were assembled and manually curated using SeqMan v5.03 (DNASTAR, USA). All genomes were submitted to GenBank (accessions PV807149–PV807155).

### Phylogenetic and genetic analyses

Nucleotide sequences of Myanmar-PBuVs were compared with BuV sequences from humans, primates, dogs, bats, rats, and international PBuV strains (Supplementary Table 3). Phylogenetic trees for whole genomes and *NS1*, *VP1*, and *VP2* genes were constructed in MEGA v11.0 (https://www.megasoftware.net/) using the maximum–likelihood method under the GTR+G+I model with 1,000 bootstrap replicates.

Multiple sequence alignments were performed using MAFFT through MegAlign (DNASTAR v5.03). Pairwise nucleotide and amino acid identity analyses were conducted, and unique mutations in NS1, VP1, and VP2 proteins were identified and evaluated.

### Statistical analysis

Descriptive statistics were used to assess PBuV positivity across age groups, clinical conditions, seasons, and farm locations. Associations between PBuV detection and epidemiological variables were evaluated using the chi-square test in IBM SPSS Statistics v25 (IBM Corp., USA). A p-value <0.05 was considered statistically significant, with 95% confidence interval (CI).

## RESULTS

### Overall PBuV positivity and farm-level distribution

This study involved a cross-sectional survey of PBuV across 19 pig farms in two regions of Myanmar from January to September 2023. A total of 445 rectal swab samples were collected and tested for PBuV using NS1 gene-specific nested PCR. Our findings revealed that 15.06% (67/445, 95% CI: 11.9–18.7) tested positive for PBuV (Table 1). The PBuV detection rate was high in Yangon, Myanmar. PBuV could be detected in both symptomatic and asymptomatic pigs by clinical status. PBuV positivity was highest in fattening pigs and followed by breeders, with both demonstrating statistical significance (p < 0.001). PBuV was most prevalent during winter (November to January), showing a significant correlation between PBuV positivity and the seasons (p < 0.05) (Tables 2 and 3).

**Table 1:** Description of rectal swab samples collected in this study from pig farms in Myanmar.

Month/year	Farm location	Sample	Age of pigs	Health status	Samples	PBuV-positive (%)
Jan/23	Hlegu, Yangon	Rectal swab	Suckling	Healthy	30	0
	Hlegu, Yangon	Rectal swab	Nursery	Healthy	25	0
	Hmawbi, Yangon	Rectal swab	Nursery and fattening	Diarrhea	35	0
	Hlegu, Yangon	Rectal swab	Nursery	Healthy	38	0
	Phugyi, Yangon	Rectal swab	Nursery	Healthy	20	2 (10.00)
	Hmawbi, Yangon	Rectal swab	Fattening	Healthy	50	39 (78.00)
Sep/23	Hlegu, Yangon	Rectal swab	Breeder	Healthy	10	3 (30.00)
	Hlegu, Yangon	Rectal swab	Breeder	Diarrhea	10	0
	Hlegu, Yangon	Rectal swab	Fattening	Healthy	10	2 (20.00)
	Hlegu, Yangon	Rectal swab	Fattening	Diarrhea	10	3 (30.00)
	Hmawbi, Yangon	Rectal swab	Fattening	Healthy	10	0
	Hmawbi, Yangon	Rectal swab	Nursery	Healthy	20	0
	Hmawbi, Yangon	Rectal swab	Nursery	Healthy	20	0
	Hmawbi, Yangon	Rectal swab	Nursery	Healthy	12	0
Jan/23	Tatkon, Nay Pyi Taw	Rectal swab	Fattening	Healthy	30	8 (26.70)
	Tatkon, Nay Pyi Taw	Rectal swab	Fattening	Diarrhea	25	1 (4.00)
	TaungNyo, Nay Pyi Taw	Rectal swab	Suckling and nursery	Diarrhea	40	9 (22.50)
	Tatkon, Nay Pyi Taw	Rectal swab	Nursery	Healthy	30	0
	Tatkon, Nay Pyi Taw	Rectal swab	Suckling	Healthy	20	0
Total					445	67 (15.06)

*Suckling = <4 Weeks, Nursery = 5–8 Weeks, Fattening = 9–20 Weeks, Breeder = Gilt, Sow, Boar, PBuV = Porcine bufavirus.

**Table 2:** Occurrence of PBuVs by age group, clinical status, and season in pig farms in Myanmar.

Category	Subcategory	Age/duration	Number of samples	Positive PBuV (%)
Age group	Suckling	<4 weeks	65	0
	Nursery	5–8 weeks	215	11 (5.12%)
	Fattening	9–20 weeks	145	53 (36.55%)
	Breeder	Boar, gilt, sow	20	3 (15.00%)
Clinical status	Healthy	—	325	54 (16.62%)
	Gastroenteritis signs	—	120	13 (10.83%)
Season	Winter	November–January	343	59 (17.20%)
	Summer	February–May	0	0
	Rainy	June–October	102	8 (7.84%)
Total	—	—	445	67 (15.06%)

PBuV = Porcine bufavirus

**Table 3:** Statistical analysis of the association between PBuV positivity and clinical status, age of pigs, and season.

Variables	Number of positive samples	Number of negative samples	p-value
Clinical status			
Healthy	54	271	0.138
Gastroenteritis signs	13	107	
Age			
Suckling (<4 weeks)	0	65	0.001**
Nursery (5–8 weeks)	11	204	
Fattening (9–20 weeks)	53	92	
Breeder (gilt, sow, or boar)	3	17	
Season			
Winter (Nov–Jan)	59	284	0.01*
Summer (Feb–May)	0	0	
Rainy (Jun–Oct)	8	94	

* Statistical significance, p < 0.05, p < 0.01, PBuV = Porcine bufavirus

### WGS of Myanmar-PBuVs

We successfully sequenced the whole genomes of seven PBuVs. The whole-genome sequences of PBuVs (n = 7) were deposited in the GenBank database under accession numbers PV807149–PV807155 (Table 4). As expected, the PBuV genome structure comprises three main proteins, including a nonstructural protein (NS1) and two structural proteins (VP1 and VP2).

**Table 4:** Description of the Myanmar-PBuVs characterized in this study.

Sample ID	Province/State	Location	Date	Age*	Health status	Sequencing	Accession #
CU34277R	Nay Pyi Taw	Myanmar	Jan-23	10 wk	Diarrhea	WGS	PV807149
CU34347R	Nay Pyi Taw	Myanmar	Jan-23	8 wk	Diarrhea	WGS	PV807150
CU34571R	Yangon	Myanmar	Jan-23	9 wk	Healthy	WGS	PV807151
CU34668R	Yangon	Myanmar	Jan-23	9 wk	Healthy	WGS	PV807152
CU34677R	Yangon	Myanmar	Jan-23	9 wk	Healthy	WGS	PV807153
CU34688R	Yangon	Myanmar	Jan-23	9 wk	Healthy	WGS	PV807154
CU34706R	Yangon	Myanmar	Sep-23	20 wk	Diarrhea	WGS	PV807155

* Suckling = <4 Weeks, Nursery = 5–8 Weeks, Fattening = 9–20 Weeks, Breeder = Gilt, Sow, Boar, PBuV = Porcine bufavirus, WGS = Whole-genome sequencing

### WGS phylogenetic analysis

**Figure 2:**
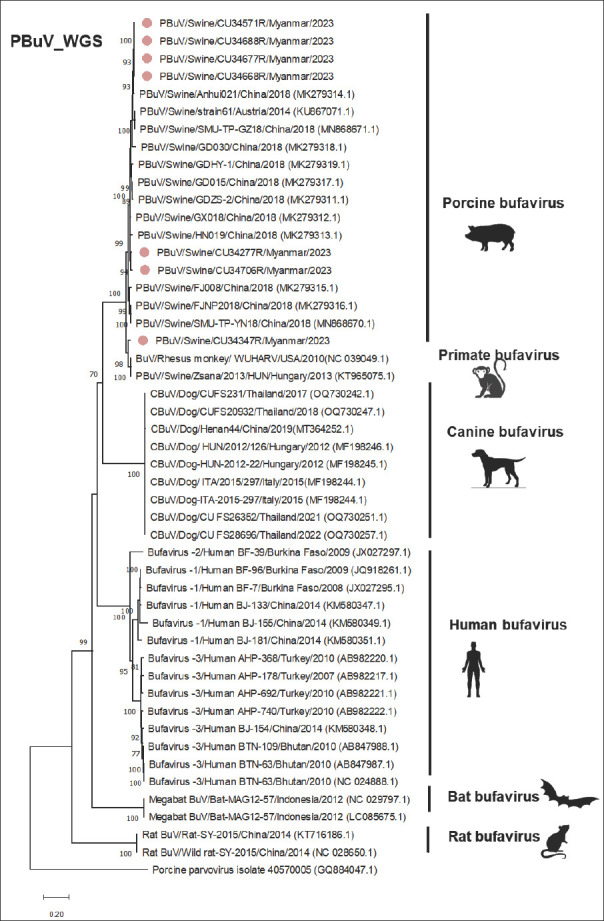
Phylogenetic tree of the whole genome of PBuVs, constructed using the maximum likelihood method, with the general time reversible model (GTR+G+I) and 1000 bootstrapping replicates. Bootstrap values of ≥ 70% are shown on the tree branches. Pink circles represent Myanmar-PBuVs. The scale bar represents 0.20 nucleotide substitutions per site.

Phylogenetic analysis of whole-genome sequences revealed that the Myanmar-PBuVs clustered with BuVs from pigs, forming a distinct grouping of PBuVs that was separated from those of dogs, rats, bats, and humans, as supported by a high bootstrap value of >70% (Figure 2). Pairwise comparisons revealed that the Myanmar-PBuV (CU34571) exhibited high nucleotide identities among them (96.69%–100%), except for CU34347 (91.81%). Myanmar-PBuV (CU34571) also showed high nucleotide identities to PBuVs from Austria (strain 61; 97.84%) and China (Anhui 021; 98.59%). In contrast, the nucleotide identities between Myanmar-PBuVs and BuV from other hosts (including humans, dogs, bats, and rats) ranged from 48.36% to 63.82% (Table 5).

**Table 5:** Nucleotide (nt) and amino acid (aa) identities of whole Myanmar-PBuV genomes with reference BuV strains.

Virus	Host	Accession number	Country	Year	WGS% nt (% aa)	NS1% nt (% aa)	VP1% nt (% aa)	VP2% nt (% aa)
This study								
CU34571R	Swine	PV807151	Myanmar	2023	100.00 (100.00)	100.00 (100.00)	100.00 (100.00)	100.00 (100.00)
CU34277R	Swine	PV807149	Myanmar	2023	97.00 (95.06)	98.27 (97.44)	98.00 (98.31)	98.24 (99.02)
CU34347R	Swine	PV807150	Myanmar	2023	91.81 (86.57)	94.35 (90.83)	90.85 (89.55)	89.06 (87.50)
CU34668R	Swine	PV807152	Myanmar	2023	99.88 (99.73)	99.70 (99.36)	100.00 (100.00)	100.00 (100.00)
CU34677R	Swine	PV807153	Myanmar	2023	99.95 (99.91)	99.88 (99.78)	100.00 (100.00)	100.00 (100.00)
CU34688R	Swine	PV807154	Myanmar	2023	100.00 (100.00)	100.00 (100.00)	100.00 (100.00)	100.00 (100.00)
CU34706R	Swine	PV807155	Myanmar	2023	96.69 (95.33)	97.58 (95.70)	97.85 (98.16)	97.72 (98.24)
Reference PBuVs								
Zsana	Swine	NC043446	Hungary	2013	92.27 (87.29)	94.67 (91.90)	90.32 (88.79)	89.20 (87.89)
GD030	Swine	MK279318	China	2018	96.57 (94.36)	94.92 (91.26)	97.49 (96.77)	97.60 (96.88)
Anhui021	Swine	MK279314	China	2018	98.59 (97.05)	99.11 (98.29)	97.90 (98.16)	97.79 (98.44)
Strain 61	Swine	KU867071	Austria	2014	97.84 (96.14)	97.34 (96.38)	98.47 (99.23)	98.45 (99.61)
SMU-TP-GZ18	Swine	MN868671	China	2018	97.84 (96.14)	97.34 (96.38)	98.47 (99.23)	98.45 (99.61)
HN019	Swine	MK279313	China	2018	96.70 (95.07)	97.40 (96.37)	98.11 (97.54)	98.19 (97.66)
GX018	Swine	MK279312	China	2018	97.41 (95.97)	97.77 (96.16)	99.24 (98.92)	99.68 (99.61)
GDHY-1	Swine	MK279319	China	2018	96.16 (93.46)	97.60 (95.94)	96.86 (96.30)	97.19 (97.06)
GD015	Swine	MK279317	China	2018	96.55 (94.09)	97.53 (95.95)	97.70 (98.16)	97.92 (98.63)
GDZS-2	Swine	MK279311	China	2018	96.19 (93.55)	97.41 (95.31)	97.07 (96.93)	97.13 (97.07)
FJNP2018	Swine	MK279316	China	2018	94.20 (90.24)	97.84 (96.16)	92.63 (90.92)	91.37 (89.65)
SMU-TP-YN18	Swine	MN868670	China	2018	94.20 (90.24)	97.84 (96.16)	92.63 (90.92)	91.37 (89.65)
FJ008	Swine	MK279315	China	2018	94.62 (90.69)	97.78 (96.38)	93.52 (91.85)	92.53 (90.82)
Human, canine, bat, and rat BuV								
BF-96	Human	JQ918261	Burkina Faso	2009	61.71 (54.93)	55.89 (47.09)	68.92 (70.23)	68.27 (69.22)
BF-7	Human	JQ027295	Burkina Faso	2008	61.72 (54.65)	55.67 (46.88)	69.00 (69.92)	68.37 (69.02)
BJ-133	Human	KM580347	China	2014	61.75 (54.65)	56.42 (48.17)	68.07 (70.23)	67.39 (69.02)
BTN-63	Human	NC-024888	Bhutan	2010	60.48 (54.04)	56.01 (48.60)	66.63 (69.04)	65.47 (67.71)
AHP-740	Human	AB982222	Turkey	2010	60.39 (53.76)	55.45 (47.74)	66.71 (69.89)	65.67 (67.51)
BF-39	Human	JX027297	Burkina Faso	2009	61.01 (53.20)	55.68 (48.17)	67.74 (69.35)	67.36 (68.49)
CU-FS231	Dog	OQ730242	Thailand	2017	63.77 (56.41)	63.36 (57.30)	64.69 (69.77)	63.45 (69.76)
CU-FS20932	Dog	OQ730247	Thailand	2018	63.95 (56.61)	63.46 (57.52)	64.86 (69.92)	63.77 (69.96)
CU-FS26352	Dog	OQ730251	Thailand	2021	63.77 (56.61)	63.26 (57.30)	64.78 (69.77)	63.55 (69.76)
CU-FS28696	Dog	OQ730257	Thailand	2022	63.82 (56.61)	63.26 (57.30)	64.86 (69.92)	63.66 (69.96)
HUN-2012-126	Dog	MF198246.1	Hungary	2012	63.81 (56.51)	63.46 (57.74)	64.69 (69.46)	63.45 (69.57)
ITA-2015-297	Dog	MF198244	Italy	2015	63.77 (56.51)	63.26 (57.30)	64.78 (69.61)	63.55 (69.57)
MAG12-57	Bat	LC085675	Indonesia	2012	58.24 (52.35)	59.08 (49.89)	59.98 (63.11)	57.45 (61.46)
SY-2015	Rat	NC-028650	China	2014	48.36 (47.08)	56.69 (50.33)	43.94 (53.57)	41.84 (52.26)

WGS = Whole-genome sequencing, NS1 = Nonstructural protein, VP1 and VP2 = Viral structural proteins, nt = Nucleotide, aa = Amino acid, PBuV = Porcine bufavirus

### Gene-specific phylogenetic analysis (*NS1, VP1*, and *VP2*)

**Figure 3:**
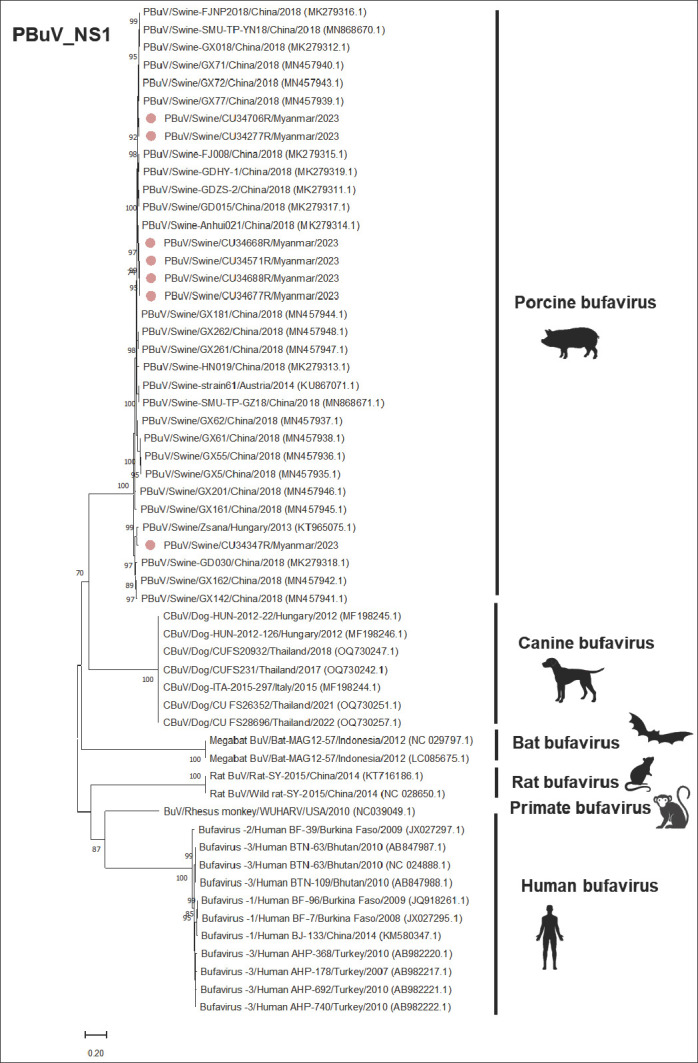
Phylogenetic tree of the NS1 of PBuVs, constructed using the maximum likelihood method, with the general time reversible model (GTR+G+I) and 1000 bootstrapping replicates. Bootstrap values of ≥ 70% are shown on the tree branches. Pink circles represent Myanmar-PBuVs. The scale bar represents 0.20 nucleotide substitutions per site.

Phylogenetic analysis of the *NS1* gene revealed that the Myanmar-PBuVs (n = 7) also clustered with PBuVs from Austria, China, and Hungary, with bootstrap values of 92%–96% (Figure 3). The nucleotide comparison of NS1 also showed that Myanmar-PBuVs had high nucleotide identities with those from Austria (strain 61), China (Anhui 021), and Hungary (Zsana), ranging from 94.67% to 99.11%. Conversely, lower nucleotide identities were observed with BuV from other species, including rats, bats, humans, and dogs, which ranged from 55.45% to 63.46% (Table 5).

**Figure 4:**
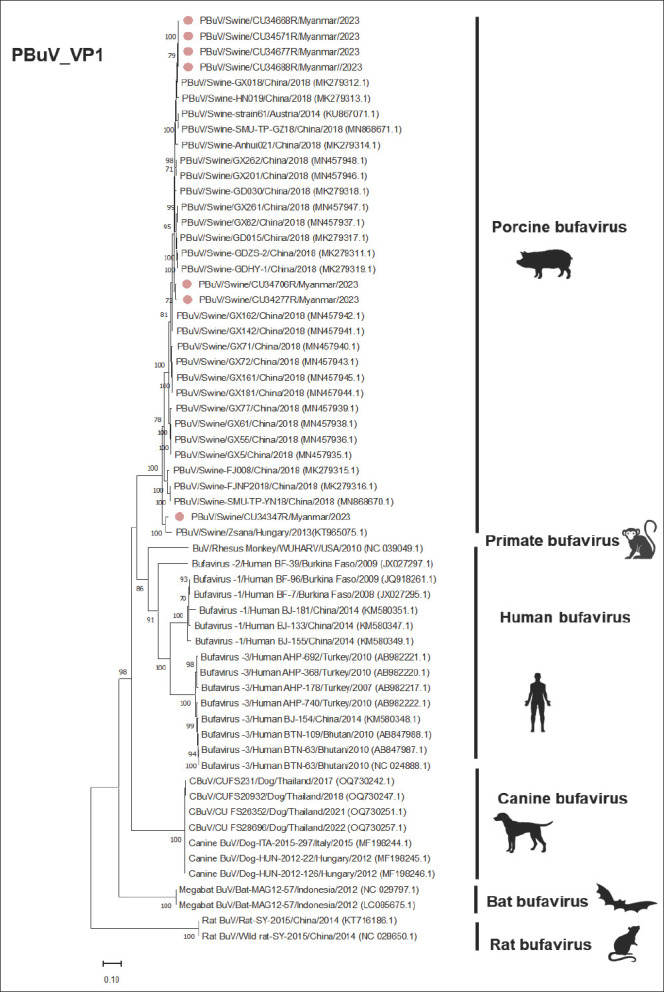
Phylogenetic tree of the VP1 of PBuVs, constructed using the maximum likelihood method, with the general time reversible model (GTR+G+I) and 1000 bootstrapping replicates. Bootstrap values of ≥ 70% are shown on the tree branches. Pink circles represent Myanmar-PBuVs. The scale bar represents 0.20 nucleotide substitutions per site.

**Figure 5:**
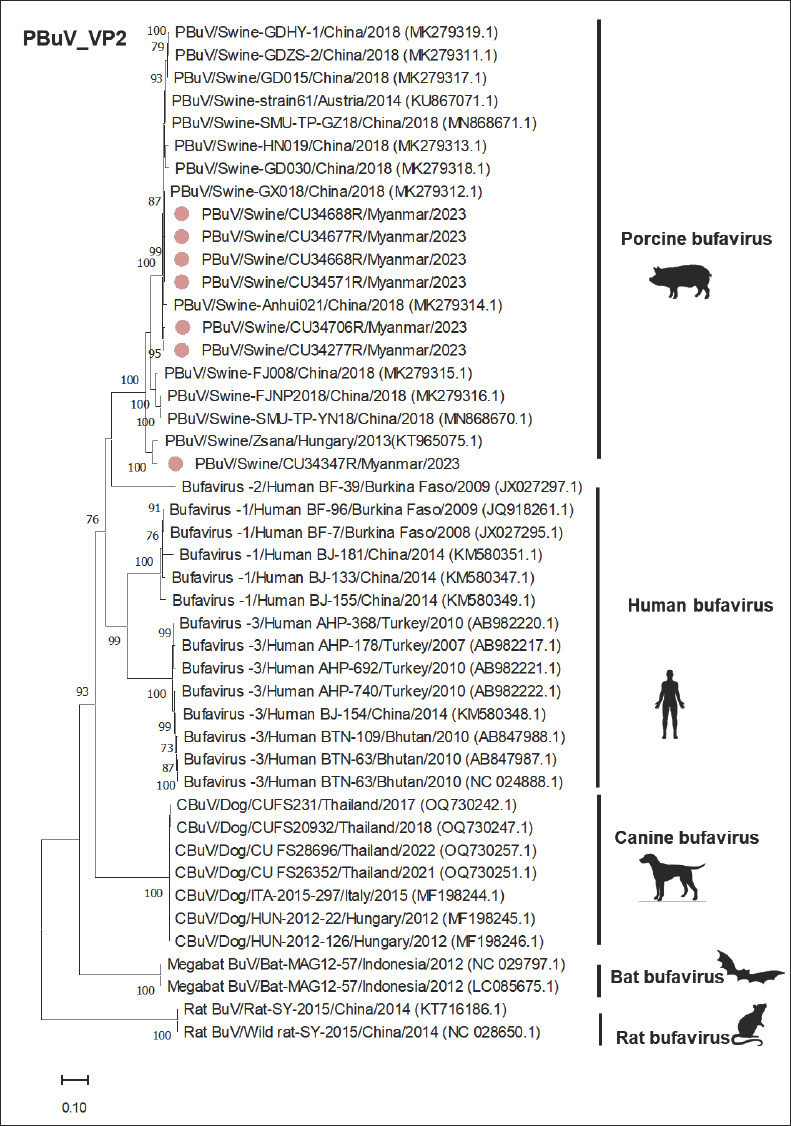
Phylogenetic tree of the VP2 of PBuVs, constructed using the maximum likelihood method, with the general time reversible model (GTR+G+I) and 1000 bootstrapping replicates. Bootstrap values of ≥ 70% are shown on the tree branches. Pink circles represent Myanmar-PBuVs. The scale bar represents 0.20 nucleotide substitutions per site.

For the *VP1* and *VP2* genes, phylogenetic analyses revealed that the Myanmar-PBuVs (n = 7) were closely related to PBuVs from Austria, China, and Hungary, with a high bootstrap value of >80%, consistent with the results of the *NS1* gene analysis (Figures 4 and 5). In the *VP1* nucleotide comparison, the Myanmar-PBuVs showed high nucleotide identities with PBuVs from Austria and China, ranging from 92.63% to 99.24%. Conversely, lower identities were found with BuVs from rats, bats, dogs, and humans. For nucleotide comparison of the *VP2* gene, the Myanmar-PBuVs exhibited nucleotide identities ranging from 91.37% to 99.68% with the Austrian and Chinese PBuVs. In contrast, BuVs from rats, bats, dogs, and humans had lower nucleotide identities (Table 5).

### Genetic and protein motif analysis

Genetic analysis of the *NS1* gene revealed that Myanmar-PBuVs contained conserved replication initiator motifs (GLHIHCLLQ and ITKYFLQKSP), identical to those found in Austrian, Chinese, and Hungarian PBuVs. Additionally, conserved amino acids at the Adenosine triphosphate or Guanosine triphosphate walker loop binding motifs (GPASTGKS) were present in Myanmar-PBuVs, resembling the reference PBuVs (Table 6). Interestingly, CU34347 exhibited 12 amino acid insertions at positions 550–561 in the NS1 region that were absent in the reference PBuVs (Supplementary Figure 1).

**Table 6:** Genetic analysis of the *NS1*, *VP1*, and *VP2* genes of Myanmar-PBuVs with reference PBuV strains.

Strain	Country	Year	A	B	C	D	E
Reference strains							
Zsana	Hungary	2013	GPASTGKS	GLHIHCLLQ; ITKYFLQKTP	HDLEY	YLGPGN	GGKGGGGGGGGGSGVG
Strain 61	Austria	2014	GPASTGKS	GLHIHCLLQ; ITKYFLQKSP	HDLEY	YLGPGN	GGKGGGGGGGGGSGVG
FJ008	China	2018	GPASTGKS	GLHIHCLLQ; ITKYFLQKSP	HDLEY	YLGPGN	AGKGGGGGGGGGSGVG
Anhui021	China	2018	GPASTGKS	GLHIHCLLQ; ITKYFLQKSP	HDLEY	YLGPGN	GGKGGGGGGGGGSGVG
SMU-TP-YN18	China	2018	GPASTGKS	GLHIHCLLQ; ITKYFLQKSP	HDLEY	YLGPGN	AGKGGGGGGGGGSGVG
FJNP2018	China	2018	GPASTGKS	GLHIHCLLQ; ITKYFLQKSP	HDLEY	YLGPGN	AGKGGGGGGGGGSGVG
GD015	China	2018	GPASTGKS	GLHIHCLLQ; VTKYFLQKSP	HDLEY	YLGPGN	GGKGGGGGGGGGSGVG
GD030	China	2018	GPASTGKS	GLHIHCLLQ; ITKYFLQKTP	HDLEY	YLGPGN	GGKGGGGGGGGGSGVG
GDZS-2	China	2018	GPASTGKS	GLHIHCLLQ; VTKYFLQKSP	HDLEY	YLGPGN	GGKGGGGGGGGGSGVG
GDHY-1	China	2018	GPASTGKS	GLHIHCLLQ; ITKYFLQKSP	HDLEY	YLGPGN	GGKGGGGGGGGGSGVG
This study							
CU34277R	Myanmar	2023	GPASTGKS	GLHIHCLLQ; ITKYFLQKSP	HDLEY	YLGPGN	GGKGGGGGGGGGSGVG
CU34347R	Myanmar	2023	GPASTGKS	GLHIHCLLQ; ITKYFLQKTP	HDLEY	YLGPGN	AGKGGGGGGGGGSGVG
CU34571R	Myanmar	2023	GPASTGKS	GLHIHCLLQ; ITKYFLQKTP	HDLEY	YLGPGN	GGKGGGGGGGGGSGVG
CU34668R	Myanmar	2023	GPASTGKS	GLHIHCLLQ; ITKYFLQKSP	HDLEY	YLGPGN	GGKGGGGGGGGGSGVG
CU34677R	Myanmar	2023	GPASTGKS	GLHIHCLLQ; ITKYFLQKSP	HDLEY	YLGPGN	GGKGGGGGGGGGSGVG
CU34688R	Myanmar	2023	GPASTGKS	GLHIHCLLQ; ITKYFLQKSP	HDLEY	YLGPGN	GGKGGGGGGGGGSGVG
CU34706R	Myanmar	2023	GPASTGKS	GLHIHCLLQ; ITKYFQQKSP	HDLEY	YLGPGN	GGKGGGGGGGGGSGVG

A = NS1 dNTP walker loop motif, B = NS1 two conserved replication initiation motifs, C = VP1 phospholipase A2 catalytic residues, D = VP1 calcium-binding loop, E = VP2 glycine-rich sequences, NS1 = Nonstructural protein, VP1 and VP2 = Viral structural proteins, PBuV = Porcine bufavirus

The N-terminus of the *VP1* gene contained phospholipase A2 catalytic residues (HDLEY) and a calcium-binding loop (YLGPGN), consistent with reference PBuVs. Genetic analysis of the *VP2* gene revealed a glycine-rich sequence (GGKGGGGGGGGGGGSGVG) at the N-terminus of the *VP2* gene in Myanmar-PBuVs, similar to that in reference PBuVs (Table 6). Variations in certain amino acids of the *VP2* gene were observed in Myanmar-PBuVs. The Myanmar-PBuVs contained some unique amino acid substitutions compared with other reference PBuVs (Supplementary Table 2).

## DISCUSSION

### Emergence and gobal distribution of BuV

Infection by BuV in pigs, specifically PBuV, was first detected in Hungary in 2016 and has since been reported in several countries. However, PBuVs had not been documented in Myanmar until now. This study highlights the first detection of PBuVs in Myanmar pigs. Our molecular survey revealed a positive PBuV rate of 15.06% (67/445). These findings were consistent with those of previous studies in Austria (13.3%) and China (16.7%), where PBuV was identified in fecal and serum samples from pigs [2, 21]. In contrast, a study conducted in Guangxi Province, China, reported a higher positivity rate of PBuV in intestinal tissues and rectal swabs of pigs, at 29.13% [22].

### Tissue tropism and clinical association

A previous study in China reported the detection of PBuV in various sample types, including rectal swabs, fecal samples, intestinal tissue, lung, spleen, and kidney, as well as serum, highlighting the broad tissue tropism of the virus [22]. Another study in China reported that PBuV was found in both serum (14%) and fecal samples (25%) [21]. PBuV was detected in both symptomatic and asymptomatic pigs in this study. The occurrence of PBuV was higher in asymptomatic pigs (16.62%, 54/325) than in symptomatic pigs (10.83%, 13/120), with no statistically significant difference (p < 0.138). Our results agreed with a study in Austria, which reported that PBuVs were detected in both healthy and diarrheic pigs, but the difference was not statistically significant [2]. However, our result contradicts a previous study conducted in Hungary, which found that PBuV was higher in pigs with posterior paralysis (90.5%) than in clinically healthy pigs (33.3%) [20]. This observation is explained by the fact that the target sampling was conducted in pigs infected with PBuVs in that previous study.

### Age-related and seasonal distribution patterns

When analyzed by age group, PBuV infection was higher in fattening pigs and breeders than in suckling and nursery pigs. This observation contradicted previous studies that detected PBuV in pigs aged 6–10 weeks in Austria and 3-month-olds in Hungary, sometimes associated with posterior paraplegia in finisher pigs [2, 20]. In this study, we observed the occurrence of PBuV across seasons, with the highest detection rates in winter. Our results showed a statistically significant difference in PBuV positivity by season. This finding was consistent with previous research on BuVs in humans and dogs in Thailand, although without statistical significance [6, 17]. However, further investigations are necessary to explore the effects of seasonal factors on BuV circulation.

### Expansion of the global genome database and evolutionary relationships

Currently, only 13 complete genomes and 16 partial gene sequences of PBuVs are available in GenBank. Our research expanded the nucleotide sequence information of the whole-genome sequences of seven Myanmar-PBuVs, which will help expand the global PBuV sequence database. Phylogenetic analysis of the whole-genome indicated that Myanmar-PBuVs belonged to the PBuV group within the *Protoparvovirus* genus and demonstrated close relationships with Austrian (strain 61) and Chinese (Anhui021, HN019) PBuVs. Note that Myanmar-PBuVs likely share a common ancestor with PBuVs from these countries. Furthermore, phylogenetic analysis of the *NS1* gene suggested that Myanmar-PBuVs were closely linked to BuV in dogs, implying a potential for interspecies transmission. Notably, there is no strong evidence of cross-species transmission of PBuV to humans. However, in a previous study, the macaque BuV was found to recombine with human BuV, suggesting its potential for cross-species transmission [13]. A previous study reported that PBuVs and human BuVs shared a recent common ancestor based on evolutionary analysis [2]. Phylogenetic analyses of the *VP1* and *VP2* genes also revealed a close relationship between Myanmar-PBuVs and human BuV, underlining the potential risk to humans of this virus, consistent with findings from previous studies [21]. Our results made a unique contribution to One Health virology and served as an early warning signal of potential zoonotic crossover.

### Genetic features and conserved motifs

Genetic analysis of Myanmar-PBuVs showed that the *NS1* gene contained two conserved replication initiation motifs (GLHIHCLLQ and ITKYFLQKSP) and a helicase motif walker (GPASTGKS). In this study, analysis of the *NS1* coding region of CU34347 showed 12 unique amino acid insertions compared with the *NS1* of the reference PBuV sequences. Notably, the NS1 protein plays a key role in viral infection and replication; thus, this amino acid insertion change in the NS1 protein may influence the virus’s virulence [25]. Additional research is needed to investigate the significance of insertions in the NS1 protein in relation to viral evolution and host adaptation. We identified highly conserved amino acid motifs in the VP1 region, including the Ca^2+^ binding loop (YLGPGN) and the catalytic center (HDLEY) of the phospholipase A2 (PLA2) motif. This result aligned with previous studies that have documented the significant roles these two conserved motifs may play in viral entry into host cells [26]. We also identified a glycine-rich motif in the N-terminus of the *VP1*/*VP2* gene that could be associated with parvovirus cellular entry. The *VP2* gene encodes a significant structural protein (VP2) that acts as an immunogen.

### VP2 gene diversity and implications for classification

BuVs found in humans can be divided into three genotypes (BuV1–3) based on the *VP1* and *VP2* sequences [27]. In contrast, CBuV found in dogs can be divided into two subgroups (A and B) [17]. To the best of our knowledge, there is no documentation on the PBuV classification. Myanmar-PBuVs showed different amino acid substitutions in the *VP2* gene analysis. For example, CU34277, CU34571, CU34668, CU34677, and CU34688 exhibited amino acid substitutions similar to those found in the Austrian PBuV strain (strain 61). In contrast, CU34347 demonstrated a similar substitution pattern to the Hungarian PBuV strain Zsana, whereas CU34706 displayed amino acid substitution patterns typical of the Chinese PBuV strains Anhui021 and GD030. Thus, the diversity of the *VP2* gene of Myanmar-PBuVs may be used for the classification of PBuVs in the future. A previous study reported that high mutations in the *VP2* gene can influence the antigenicity and genetic diversity of parvoviruses [28]. Further investigations are needed to determine whether VP2 amino acid changes affect PBuV biological properties and classification.

### Unique amino acid substitutions and research implications

Myanmar-PBuVs exhibited 25 unique amino acid substitutions compared with the reference PBuV strains (strain 61, Zsana, Anhui021). This observation supported earlier research from China, which identified 25 amino acid variations in PBuVs, including two unique changes in the *VP2* sequence analysis [22]. Previous studies have shown the presence of PBuVs in both healthy and diarrheic pigs; however, the pathogenesis and its link to diarrhea remain unclear [20]. Additionally, a survey in China highlighted the lack of suitable animal models and cell culture systems necessary to investigate the disease mechanism and pathogenicity of BuV in animals [21]. Our findings on the novel amino acid insertions in *NS1* (in CU34347) and *VP2* substitutions provided evidence of possible PBuV evolution specific to the region, which had not been previously reported.

## CONCLUSION

This study provides the first molecular and genomic evidence of PBuV circulating in pig farms in Myanmar, with an overall positivity rate of 15.06% (67/445). Detection across pigs of different age groups, particularly the high prevalence in fattening pigs, and the marked seasonal trend with the highest positivity during winter, indicate active viral circulation within production systems. WGS of seven Myanmar-PBuV strains expanded the global genomic database and revealed close phylogenetic relatedness to PBuVs from Austria and China, suggesting potential regional or transboundary viral movement. The discovery of unique amino acid insertions in the NS1 protein and distinct VP2 substitutions further points to possible micro-evolutionary changes occurring within Myanmar pig populations. Although Myanmar-PBuVs remained genetically distinct from human, canine, bat, and rat BuVs, the close clustering with human BuV in *VP1* and *VP2* phylogenies underscores a potential zoonotic risk that warrants One Health–based surveillance.

From a practical standpoint, these findings highlight the need for improved virological monitoring in Myanmar’s pig industry, especially in fattening farms and during high-risk seasons. Strengths of the study include systematic cross-sectional sampling across major pig-producing regions, comprehensive genomic characterization, and multi-gene phylogenetic analyses that provide a detailed understanding of PBuV diversity and evolutionary positioning.

However, this study had limitations. Sample representation was restricted to farms willing to participate, resulting in uneven sampling across age groups and seasons. The exclusive focus on PBuV detection did not allow assessment of coinfections with other enteric viruses, nor was disease causality investigated. Constraints in cell culture systems and the absence of experimental infection models also limited the understanding of PBuV pathogenicity.

Future research should focus on broader and longitudinal sampling, multiplex pathogen screening, and clinical follow-up to clarify the pathogenic role of PBuV. Functional studies on *NS1* and *VP2* mutations are essential to determine their impact on viral evolution, virulence, and host adaptation. Investigations into environmental persistence, transmission dynamics, and cross-species spillover potential will further strengthen One Health preparedness.

In conclusion, this study establishes a foundational understanding of PBuV epidemiology and genomic characteristics in Myanmar and signals the importance of continuous surveillance. The detection of genetically diverse PBuV strains, along with potential zoonotic relevance, underscores the need for integrated veterinary and public health efforts to monitor, manage, and mitigate emerging BuV threats in Southeast Asia.

## DATA AVAILABILITY

The authors declare that the data supporting the findings of this study are available in the supplementary tables. The nucleotide sequence data that support the findings of this study are openly available in the GenBank database at https://www.ncbi.nlm.nih.gov/genbank/ under accession numbers PV807149–PV807155. Data availability on pig farms, pig sample details, and sequence alignments are available upon request from the corresponding author.

## AUTHORS’ CONTRIBUTIONS

HWP, KU, EMP, YNT, TNL, and MTM: Sample collection. HWP, KC, CN, and SC: Virus detection, whole-genome characterization, and phylogenetic analysis. KC, CN, EMP, and SC: Phylogenetic analysis. HWP: Drafted the manuscript. AA: Designed the study, data analysis, and drafted and revised the manuscript. All authors read and approved the final version of the manuscript.
